# Improving gender data is essential for progress on equity and empowerment

**DOI:** 10.1016/j.ssmph.2019.100494

**Published:** 2019-11-20

**Authors:** Emily Courey Pryor, Papa A. Seck

**Affiliations:** a Data 2X, Washington, DC, USA; b UN Women, New York, NY, USA

Understanding and measuring women's empowerment – and acting on that information – is a necessity for global development and human rights. This fact underpins Sustainable Development Goal (SDG) 5, which seeks to “achieve gender equality and empower all women and girls.”

However, official metrics for empowerment—which are critical for monitoring progress toward SDG 5— are lacking. While previous literature has attempted to outline a conceptual framework for the term, breaking the idea down into foundational markers such as resources, voice, agency, and choice (Kabeer, 1999; van Eerdewijk et al., 2019), translating these concepts into standardized measurements is difficult, complicating the process of designing interventions and measuring results.

Of the 17 SDGs, 12 goals contain 54 indicators explicitly relating to women and girls. Gender data is necessary to measure these indicators, and yet significant gaps exist: UN Women estimates that less than 24% of available gender data was generated in the last decade, and only 22% of gender-specific indicators are produced with regularity worldwide (UN Women, 2018: 56). Of the data that is available, quality is a significant challenge; substandard data inhibits the international community from gaining a clear picture of the scope and scale of gender differences (Buvinic and Levine, 2016). These gender data gaps impede the measurement of empowerment and, in turn, hamper SDG 5 achievement.

In light of the dual challenges of producing and using quality gender data and measuring empowerment, each paper in this Special Issue of *Social Science and Medicine—Population Health* uses gender data to consider the ways in which women's empowerment mediates both gender equality and health, shedding much needed light on how improving the measurement of empowerment can contribute to better monitoring and tracking progress on SDG 5. Specifically, the set of papers in this Special Issue contribute to better SDGs monitoring in at least two ways.

First, SDGs indicators were selected for global monitoring, and thus require internationally accepted standards and methodologies for measurement and cross-national comparability. While desirable, these criteria can also hamper measurement of nationally and locally specific challenges, particularly when considering gender and intersectionality. By presenting measures of empowerment in specific contexts— from sanitation (Bisung and Elliott,) and reproductive health (Hinson et al.) to child nutrition (Jones et al.) and fertility (Samari)— and using flexible, culturally-specific definitions (Bisung and Elliott,; Hinson et al.; Jones et al.; Schuster et al.), the articles utilize gender data in innovative new ways to provide new insights and to generate ideas for additions to standardized reporting. At the same time, the need to address existing gaps in the SDG indicator framework is not lost, such as the efforts of Clark et al. to identify classes of intimate partner violence and Heise et al. to catalog psychological abuse, both of which could influence how we monitor SDG target 5.2.

In addition, while the SDG indicators are somewhat lacking in nuance, the papers in this Special Issue use data to uncover the realities of lived experience, helping to answer specific policy questions and provide solutions to policymakers. For instance, by highlighting the normative influence of gender stereotypes on public health, Baird et al. and Shakya et al. demonstrate how norms impact adolescent development, while Saikia et al. and Silverman et al. consider attitudes affecting women. Each text uses data to expose new insights about women's daily lives (particularly Clark et al.; Green et al.; Heise et al.; Klugman et al.; Reed et al.; Silverman et al.).

Taken together, the papers in this edition demonstrate how progress can be better monitored in support of SDG 5. The edition also highlights the importance of evidence rooted in lived experience—demonstrating that a commitment to gender data can help to unmask hidden inequalities and pave the way in delivering not only for SDG 5, but for the full aspirations of the Sustainable Development Goals.

At the same time, we know there are wide gaps in our knowledge still to be filled. A forthcoming Data2X brief (in press) maps gender data gaps in global health, exposing weaknesses across the field. For example, when considering women's utilization of health services, demand can be affected by social norms, an issue not captured by the SDGs (but acknowledged by Moradhav and Saikia). Another challenge is the lack of data and visibility for key population groups in global SDGs monitoring. For instance, the field of gender and health – including most papers in this special issue – continues to have a strong focus on reproductive health, meaning that issues relevant for older populations are seldom covered. While several papers relate to adolescent girls, gaps in data availability on children aged 10–14 continue to limit our knowledge. Similarly, increased focus on men, boys and masculinities could also provide much-needed insights. The way forward is in efforts to collect and, as these articles do, utilize sex-disaggregated data globally. Unfortunately, we also know that a key reason for persistent gender data gaps is the lack of resources devoted to the issue (Data2X, in press, Data2X, 2019b). Addressing these challenges, and advancing the work and insights described throughout this edition, will also require sustained efforts to finance the collection and utilization of sex-disaggregated data.

Emily Courey Pryor is Executive Director of Data2X, a technical and advocacy platform advancing the availability, quality, and use of gender data worldwide. Papa Seck is Chief Statistician and leads the Women Count programme of UN Women, the UN's official body championing global gender equality. The authors also wish to acknowledge the contributions of Natalie Cleveland from Data2X.
